# Supplementary material for: DNA methylation and single-nucleotide polymorphisms in DDX58 are associated with hand, foot and mouth disease caused by enterovirus 71
Source: PLoS Negl Trop Dis. 2022 Jan 18;16(1):e0010090. doi: 10.1371/journal.pntd.0010090 (PMC8765647; doi:10.1371/journal.pntd.0010090)
Supplement: S1 Table — (DOCX) [file pntd.0010090.s002.docx]

**S1 Table. Clinical data in 120 EV71-HFMD cases and 60 healthy control cases.**

|  | Mild, n=60 | Severe, n=60 | Control, n=60 | *P* |
| --- | --- | --- | --- | --- |
| Sample collection time | 2017~2020  (May to July, October to November  each year) | | 2017~2019  (September each year) | / |
| Onset age (month) | 41(19-74) | 25(11-53) | 35(27-66) | 0.131^a^ |
| Male/female | 34/26 | 46/14 | 40/20 | 0.067 |
| Vomiting (No/Yes) | 52/8 | 20/40 | / | <0.001 |
| Fever course (≤3/>3 days) | 18/42 | 15/45 | / | 0.540 |
| High fever (≤39/>39℃) | 28/32 | 13/47 | / | 0.004 |
| Neutrophil%, median (IQR) | 44.87(29.20-56.80) | 71.10(57.23-75.90) | / | <0.001 |
| Glucose (mmol/L), median (IQR) | 4.92(3.98-6.30) | 6.50(5.50-8.58) | / | <0.001 |
| Lymphocyte%, median (IQR) | 53.00(37.70-60.74) | 24.60(18.40-38.03) | / | <0.001 |
| *P*^a^: combination of mild and severe group vs control group. | | | | |
