# Supplementary material for: DNA methylation and single-nucleotide polymorphisms in DDX58 are associated with hand, foot and mouth disease caused by enterovirus 71
Source: PLoS Negl Trop Dis. 2022 Jan 18;16(1):e0010090. doi: 10.1371/journal.pntd.0010090 (PMC8765647; doi:10.1371/journal.pntd.0010090)
Supplement: Attachment — Submitted filename: Response to Reviewers.docx [file pntd.0010090.s007.docx]

Dear editor and reviewers,

Thank you for the invitation to revise our manuscript (ID: PNTD-D-21-00440), which are modified extensively according to the reviewer comments. The changes are highlighted in red. We reply point-by-point to those comments in the following part.

Thank you again for the opportunity to revise our manuscript, and we hope the new version can be considered suitable for publication.

Sincerely yours,

Song Zhai and Shuang-suo Dang, MD, PhD

Department of Infectious Diseases

The Second Affiliated Hospital of Xi’an Jiaotong University

157, Xiwu Road

Xi’an 710004

China

**RESPONSES TO COMMENTS FROM REVIEWER**

**Reviewer #1:**

**Methods:**

1. The authors don't mention how they classify the patients as mild vs severe. More information in this regard would be helpful.

**Author:** Thanks for your helpful suggestion. The definition of severe and mild EV71 infection has been added in the participants part of *Methods* section (line113-115, page 6).

**Results:**

2. They show that DNA methylation on the promoter of DDX58 at some of the locations were higher in the severe dataset wrt to the mild and control. While some of the places showed a clear distinction between control and severe datasets with mild being in between the two, some of the regions like CpG 248, 168 and 179 had no difference between control and severe (figure 2). It would be more helpful if the authors provide individual data points in each of the 3 subsets to better understand the range of values (in Figure 2,3,8).

**Author:** Thanks for your helpful suggestion. To better characterize the methylation of DDX58, we also assessed the methylation level of each CpG site. We observed that the 29, 134, 154, 168, 179, 232, 246 and 248 CpG sites of DDX58 had a significantly higher methylation pattern in severe HFMD patients than in the corresponding healthy controls and mild HFMD patients. For more precise and reliable conclusions, we used FDR as adjusted p value and found that 29, 134, 154, 232, 246 and 248 CpG sites of DDX58, as well as 50 and 86 CpG sites of IFIH1 were still positive sites. The details have been displayed in Revised Table 1, Table 2 and revised Figure 2A and 2B.

**Conclusions:**

3.In conclusion the authors claim that DDX58 expression and promoter methylation are associated with EV71 infection progression, which is collaborated by their data. Although their claim that the levels can be used to distinguish patients with severe disease may not hold true as the difference between control and severe patients in though significantly different in many cases was not hugely separated.

**Author:** Thanks for your helpful suggestion. In order to get more accurate results, we used FDR as adjusted p value (Revised Table 1 and Table 2) and draw the followed conclusion: DDX58 expression and the average methylation of DDX58 promoter are significantly different among the three groups, and methylation of some CpG sites are significantly different as well, such as CpG sites 29, 134, 154, 232, 246 and 248. Indeed, some CpG sites may not be able to distinguish patients with severe disease, but it is normal because not every CpG sites could plays a role in DDX58 expression and the average methylation of DDX58 promoter.

4. Line 121: uppercase W in "written"

**Author:** Thanks for the reminder, and we have revised the mistake.

**Reviewer #2:**

**Methods:**

1. Bonferroni corrections for multiple testing are needed, which should include any testing that was done on data that was not presented in the paper. In order to preserve the statistical significance of the main findings it may be necessary to clearly state a small number of hypotheses to be prospectively tested and present fewer minor findings. Maybe move them to supplementary data.

**Author:** Thanks for your helpful suggestion. We have added the Adjusted p-value (FDR) analysis according to your suggestion (Revised Table 1 and Table 2).

2. How were the 60 control children recruited?

**Author:** Thanks for your kind reminder, we have added the details about the 60 control children in *Methods* section (line120-124, page 6-7) and supplementary materials (S1 Table). The 60 control children come from the those who coming to Xi’an Jiaotong University Second Affiliated Hospital and Xi’an Children’s Hospital for pre-kindergarten health checks at the same year with the patients. We collected their blood samples with the informed consent of the children's guardians.

3. Were the 3 groups of children recruited simultaneously? What were the dates for the first and last recruited child in each group?

**Author:** Due to the seasonality of HFMD, we collected the samples of severe and mild disease patients during the peak season (May to July, October to November) from 2017 to 2020. In China, most children would be given a health checkup before entering kindergarten in September, so their blood samples were collected in September from 2017 to 2019. It took us four years to collect enough blood samples from 120 patients and 60 controls to extract RNA and DNA, and follow-up tests were conducted simultaneously.

4. A table comparing the age/sex details of the children in each group would be helpful as well as first and last dates of recruitment.

**Author:** Thanks for your kind reminder, we have added the details about the 60 control children in *Methods* section (line 120-124, page 6-7) and supplementary materials (S1 Table).

5. Give more details of the patients who were excluded. E.g. How many? What were all the reasons?

**Author:** Thanks for your kind reminder, we have added the exclusion criteria in *Methods* section (line117-119, page 6), and more details about the 120 patients and the 60 controls has been displayed in supplementary materials (S1 Table).

**Results:**

1. The units of measurement should be stated on the y axis of figures and also in the figure legends.

**Author:** Thanks for your helpful suggestion. We have revised the y axis of figures according to your suggestion.

2. How were the heat maps made? It would help to explain the meaning of the +1 and -1 values.

**Author:** Thanks for your helpful suggestion. The heatmap (“heatmap.2” R package) was constructed on the average methylation expression after standardization (-1 to +1) in order to construct separate methylation correlation matrices for disease status. The bigger the number is, the higher the methylation expression is. We have revised the methods according to your suggestion (line 189-191, page 10).

3. The word “compound” in figure 7 is inaccurate.

**Author:** Thanks for your helpful suggestion. We have revised the figures according to your suggestion, and modified “compound” to “Methylation levels”.

4. I assume that the mean/median levels of GAPDH expression were the same in the groups?

**Author:** Yes, the mean/median levels of GAPDH expression were the same in the groups, and we have added the sentence in *Methods* section (line 156-157, page 8).

5. The SNPs were genotyped by two different techniques. Which technique was used for the results in the publication? Did the two techniques give the same results?

**Author:** Thanks for your helpful suggestion. The imLDR^TM^ method determined genotypes by PCR ampliﬁcation of genomic DNA and sequencing. Therefore, the SNPs were genotyped by only imLDR^TM^ technique, and we have revised the method (line 170, page 9). We used three models (Codominant model, Dominant model and Allele model) to analyze DDX58 rs3739674 genotype distribution and allele frequencies in the mild and severe HFMD groups (Revised Table 4), and these are also used in other publication. All three models could give the same results that G base variants were more likely to be associated with severe HFMD than C base variants in DDX58 rs3739674 genotype. We apologize for any misunderstanding and ambiguity caused by this inclarity.

6. What were the results of the Hardy-Weinberg tests for each group?

**Author:** Thanks for your helpful suggest. We have tagged the results of Hardy-Weinberg tests for DDX58 rs3739674 and IFIH1 rs1990760 allele frequencies in the mild and severe HFMD groups. It is a pity that we did not perform SNP analysis in control group because it is extremely difficult to get enough blood samples from healthy children to do so much test. We would collect more samples from healthy children to do follow-up research.

7. What is the blue rectangle in the upper left of figure 1?

**Author:** The blue rectangle of Figure 1 represents severe cases, green mild cases and red healthy controls.

**Conclusions:**

1. In the conclusion of the Discussion the authors assume that the DDX58 parameters influence severe infection. It seems more likely that the infection influences the changes in methylation and expression. If DDX58 methylation was the cause of the severe infection (ie it preceded the infection) then the variance in the control group should be the same as the variance in the combined infected groups? In this data, high levels of DDX58 methylation are only seen after infection.

**Author:** Thanks for your helpful suggest. It is the limitation of this research, we could only draw a conclusion that DDX58 expression and DDX58 methylation are relevant with the severe infection because we only collected blood samples after infection, and use healthy children as controls instead of the patients themselves before infection. In this research, we could not figure out the causal relationship between DDX58 and severe HFMD, maybe follow-up animal experiment could explain.

2. How biologically significant are these findings? It would be good to discuss this. How much of the variance (r squared) in DDX58 methylation is accounted for by clinical outcome? How much of the variance in DDX58 expression is accounted for by DDX58 methylation? How much of the variance in clinical outcome is accounted for by DDX58 expression?

**Author:** Thanks for your helpful suggest. We have performed Simple linear regression analysis between DDX58 methylation and clinical outcome, DDX58 expression and DDX58 methylation. The result has been added in *Results* section (line 274-278, page 13-14). Simple linear regression analysis shows the variance in DDX58 expression accounted by HFMD severity (R^2^=0.293, p<0.001), by DDX58 methylation (R^2^=0.058, p=0.008), and the variance in IFIH1 expression accounted by HFMD severity (R^2^=0.033, p=0.046), by IFIH1 methylation (R^2^, p=0.347). The variance in DDX58 expression could be 29.3% explained by the severity of HFMD, while IFIH1 expression 3.3%, which could draw the same conclusion with the results that DDX58 expression is significantly lower in severe patients: DDX58 expression is associated with HFMD severity, but IFIH1 not. The variance in DDX58 expression accounted DDX58 methylation suggest that high DDX58 methylation may play a less role in the depression of DDX58 expression, at least not as much as the severity of HFMD. What’s more, the variants in IFIH1 expression could not be explained by IFIH1 methylation (p=0.347). In addition, we found a wrong p value in Table 3 during statistical data verification process, and we have revised and tagged that.

3. How clinically significant are the findings? Are they likely to have any influence on patient management?

**Author:** Thanks for your question. Based on this research, we could predict the severity of a EV71-HFMD patients early by testing DDX58 expression or methylation once EV71-HFMD symptoms appear, and potential severe EV71-HFMD patients would be treated more concisely and promptly. This research could also be the basis of follow-up research about the relationship between DDX58 and the prevention and treatment of severe EV71-HFMD.

4. Etc is not a scientific expression

**Author:** Thanks for your helpful suggestion. We have revised the manuscript according to your suggestion (line 305、line 312, page 16).

**Editorial and Data Presentation Modifications?**

1.The standard of English is generally very good, except for the Author Summary which needs re-writing.

**Author:** Thanks for your helpful suggestion. We have revised the Author Summary according to your suggestion.

**Summary and General Comments**

The data is of interest and is a logical extension of previous work from the author's laboratory. There is too much data presented. Only present data that you are actually going to discuss.

**Author:** Thanks for your helpful suggestion. We have streamlined the chart (revised Table 1-4) and figures (revised Figure 1-7).
