# Supplementary material for: DNA methylation and single-nucleotide polymorphisms in DDX58 are associated with hand, foot and mouth disease caused by enterovirus 71
Source: PLoS Negl Trop Dis. 2022 Jan 18;16(1):e0010090. doi: 10.1371/journal.pntd.0010090 (PMC8765647; doi:10.1371/journal.pntd.0010090)
Supplement: Attachment — Submitted filename: Response to reviewers.docx [file pntd.0010090.s008.docx]

Dear editor and reviewers,

Thank you for the invitation to revise our manuscript (ID: PNTD-D-21-00440), which we have modified extensively according to the reviewer comments. The changes are highlighted with red font and yellow background, and we also submitted a clean version. Below we reply point-by-point to those comments.

Thank you again for the opportunity to revise our manuscript, and we hope the new version can be considered suitable for publication.

Sincerely yours,

Song Zhai and Shuang-suo Dang, MD, PhD

Department of Infectious Diseases

The Second Affiliated Hospital of Xi’an Jiaotong University

157, Xiwu Road

Xi’an 710004

China

**RESPONSES TO COMMENTS FROM REVIEWER**

**Reviewer #1: The authors have addressed all the major comments and the revised manuscript is stronger and more clear.**

Author: Thanks for your comments and suggests, we have learned a lot.

**Reviewer #2: This manuscript contains interesting data exploring the relationships between HFMD infection severity and DDX58 and IFIH1 promoter methylation, gene expression and SNP genotypes. There are still some improvements to be made.**

Author: Thanks for your comments and suggests, based on that we have modified the manuscript extensively, and the declaration about the revise follows below.

**1. Table S1 shows there were two windows for recruiting patients. Was the number of severe and mild cases the same in each window?**

Author: Thanks for your reminder. The number of severe and mild cases recruited in this two season are about the same, without significant difference.

**2. The timing of the blood sample taking needs to be stated more clearly. Was the blood taken before or after the patients had been classified into mild and severe groups? It would also be interesting to know the percentage of hospitalised patients that would normally be expected to develop severe disease.**

Author: Thanks for your question. HFMD patients are diagnosed by clinical symptoms, which can be divided into mild and severe cases according to the presence of neurological symptoms in the outpatient department. Their blood was collected in the outpatient department, which means after classification. Maybe there is few mild to severe cases, but we have not observed.

**3. Dengue virus infections have recently been reported in Xi’an and the peak of dengue infections overlaps HFMD. Is it possible that there was a different frequency of subclinical dengue infections between the mild and severe groups that might have influenced the study parameters?**

Author: Thanks for your reminder. We have investigated and confirmed the incidence of dengue fever in Shaanxi Province, which indicated that Xi 'an is not a dengue epidemic area (The incidence is about 0.02 per 100,000) [1], so it is few possible that subclinical dengue infections would influence the severity of HFMD or other relevant parameters.

[1] https://www.phsciencedata.cn/Share/ky_sjml.jsp?id=f49cd1ba-2715-4205-93ec-a0085af4c581.

**4. Lines 226/227 say the 86 CpG has the highest methylation level in the severe group, but the data in the heat map (1C) and Table 2 show the opposite. This needs explanation.**

Author: Thank you for pointing out this expression mistake. We have described the expression of these two sites respectively and modified it in line 229-232.

**5. The average methylation rectangles in heat map 1C clearly show that the mild group (brown) has higher methylation than the other groups (blue). However, the data in figure 1D and in Table 2 do not show this. Why not?**

Author: Thanks for your reminder. We have checked the data and confirmed that IFIH1 methylation in mild group is higher than that in the severe group (Fig 1C) but without significant difference (p>0.05), so we didn’t show that in Fig 1D and Table 2.

**6. Line 188. Say ‘the significance of differences between the groups was analysed ……’**

Author: Thank you for pointing out our mistake. The expression has been modified in line 189.

**7. CpG 179 had the highest methylation in the healthy group, not the severe group.**

Author: Thanks for your comment. The CpG 179 was indeed the highest in the healthy group. And it was corrected in line 223-224.

**8. It is hard to imagine how a difference of between 7 and 9 methylated cytosines per 1000 cytosines (Table 1) can be of biological significance, although the data do show this difference is statistically significant. This needs to be discussed.**

Author: Thanks for your question. Indeed, we also agree the significant differences of the CpG methylation don’t mean the whole methylation or expression, not even biological significance. We have performed Simple linear regression analysis between DDX58 methylation and clinical outcome, DDX58 expression and DDX58 methylation. The result has been added in Results section (line 277-281).

Simple linear regression analysis shows the variance in DDX58 expression accounted by HFMD severity (R2=0.293, p<0.001), by DDX58 methylation (R^2^=0.058, p=0.008), and the variance in IFIH1 expression accounted by HFMD severity (R^2^=0.033, p=0.046), by IFIH1 methylation (R2, p=0.347). The variance in DDX58 expression accounted DDX58 methylation suggest that high DDX58 methylation may play a less role in the depression of DDX58 expression, at least not as much as the severity of HFMD. What’s more, the variants in IFIH1 expression could not be explained by IFIH1 methylation (p=0.347).

**9. Is the text in the figures large enough to be legible in the published article?**

Author: Thanks for your reminder. We have enlarged the text in the figures.

**10. Does the low lymphocyte count in the severe group account for the methylation and mRNA transcription differences? That is, do lymphocytes have lower methylation levels and higher mRNA levels than non-lymphocytes and so when the lymphocyte count falls it appears as if methylation has increased and mRNA has decreased?**

Author: Thanks for your reminder. We have performed analysis about the relation between DDX58 methylation and Neutrophil/ Lymphocyte in Table 3, which could only declare the correlation of them not the causal relationship. Our conclusion is that high DDX58 methylation are always accompanied with low lymphocyte, not lymphocytes have lower methylation levels and higher mRNA levels than non-lymphocytes. We apologized for the misunderstanding.

**11. Do methylation levels or mRNA levels make better predictions of disease severity than the lymphocyte count? If you were looking for a clinically useful prediction assay the lymphocyte count might be more convenient.**

Author: Thanks for your suggestion. Actually, we have done the research about the prognostic value of neutrophil-to-lymphocyte ratio in predicting death risk in HFMD patients. However, we don't think lymphocytes are a viable predictor of disease severity, because the changes of lymphocytes happen after HFMD disease more possibly than before the disease. The clinical manifestations of patients infected with the same pathogen EV71 were diversified in different children, suggesting that the body manifestations after infection were not only related to viral factors, but also to host factors. Our research focused on the innate immunity of the host to explore the expression, methylation and the possibility of mechanism how severe HFMD happens.

**12. Are blood leucocytes, a complex mixture of many cell types, a good choice for these analyses? Surely it should be the cells infected by the virus. Maybe in future work you could determine which leucocytes these changes were occuring in. This would be relevant to understanding pathophysiology.**

Author: Thanks for your question. We have mentioned lymphocyte changes are more likely to occur after viral infection because viral infection triggers an immune response in question11. Maybe DDX58/IFIH1 plays a role in HFMD through blood leucocyte given the correlation between them, so we would design experimental proofs later.

**13. Since the distributions of the methylation and gene expression data were not normal, a Spearman rank order correlation coefficient is more appropriate.**

Author: Thank you very much for pointing out the statistical mistake. We have reprocessed the data with Spearman rank correlation coefficient and modified it in line 193-194 and Fig 5.

**14. Were the results of the Students t test adjusted for non-normal distributions?**

Author: Thanks for your comment. This is a clerical error. The statistical method we use is the K-W test in the nonparametric test, and it was corrected in line 195.

**15. Line 337. What is a virus cell?**

Author: Thank you for finding the mistake, the correct expression should be “virus-infected cells”, and it was corrected in line 341.

**16. Use the word ‘children’ instead of ‘kids’.**

Author: Thanks for your comment. The word “kids” in the text have been replaced with “children” (line 49).
